# Hypertension Management in Tribal Primary Health Centers: Advancing Equity and Access

**DOI:** 10.7759/cureus.85535

**Published:** 2025-06-07

**Authors:** Sudip Bhattacharya, Om Prakash Bera, Lal Majhi, Debajit Sarkar, Pankaj Bhardwaj, Krupal J Joshi, Himel Mondal, U Venkatesh, Pradeep Aggarwal

**Affiliations:** 1 Department of Community and Family Medicine, All India Institute of Medical Sciences, Deoghar, IND; 2 Department of Non-communicable Diseases, Civil Registration, and Vital Statistics Data for Health, Global Health Advocacy Incubator, Washington, DC, USA; 3 Nodal Office of Non-communicable Diseases, National Health Mission, Jharkhand Government, Ranchi, IND; 4 State Non-communicable Disease Consultancy, World Health Organization, Ranchi, IND; 5 Department of Community and Family Medicine, All India Institute of Medical Sciences, Jodhpur, IND; 6 Department of Community and Family Medicine, All India Institute of Medical Sciences, Rajkot, IND; 7 Department of Physiology, All India Institute of Medical Sciences, Deoghar, IND; 8 Department of Community and Family Medicine, All India Institute of Medical Sciences, Gorakhpur, IND; 9 Department of Community and Family Medicine, All India Institute of Medical Sciences, Rishikesh, IND

**Keywords:** community health, developing country, health system development, high blood pressure, hypertension, jharkhand, non-communicable disease, primary care, tribal health, tribals

## Abstract

Hypertension, often called the “silent killer,” is emerging as a major public health concern in India, including among tribal populations that were once considered low-risk. In Jharkhand (a state in India), a region with a high proportion of tribal populations and predominantly rural communities, the prevalence is rising. Contributing factors include delayed diagnosis, restricted access to healthcare, limited awareness about health, and lifestyle shifts such as greater consumption of alcohol and tobacco. There are also notable differences across genders and a rising trend in obesity, particularly in areas with significant tribal presence. The state’s primary healthcare system faces multiple challenges, including poor road connectivity, workforce shortages, and cultural barriers to accessing care. Despite these challenges, Jharkhand has made significant progress through the National Programme for Prevention and Control of Non-Communicable Diseases (NCDs). Community-based screening by Accredited Social Health Activists (ASHAs), the use of e-Sanjeevani for tele-consultation, and a robust referral system have improved diagnosis and treatment linkage. Innovative outreach strategies, such as home-based clinics during tribal festivals, have potential in overcoming access barriers in remote tribal areas. To strengthen and scale these efforts, researchers have proposed the HTN-AAROGYA framework, focusing on Accessible Awareness, Routine care, Outreach, Guidance, Yield, and Assistance. This model uses digital tools and community engagement to promote culturally sensitive, decentralized hypertension management. Jharkhand’s experience offers scalable lessons for other tribal regions in India, emphasizing the need for tailored strategies, continued research, and policy-level support to address the growing NCD burden.

## Editorial

Hypertension, often dubbed the “silent killer,” has emerged as one of the most pervasive non-communicable diseases (NCDs) globally. Characterized by persistently elevated blood pressure levels, it is a major risk factor for cardiovascular diseases, stroke, renal failure, and premature mortality [1]. In India, the burden of hypertension has increased steadily over the years, affecting both urban and rural populations. However, a particularly concerning development is its growing prevalence among the country’s tribal communities, who have traditionally been perceived as low-risk due to their active lifestyles and natural diets [2]. This shift calls for immediate attention and a tailored approach to hypertension control, especially at the primary healthcare level. Jharkhand, a state in the eastern part of India, presents a compelling case in this regard. A relatively young state, it is home to around 3.2 crore people, of whom 26.21% are from Scheduled Tribes (STs) and 12.08% from Scheduled Castes (SCs). A significant majority (75.95%) of the population resides in rural and often difficult-to-reach areas [3]. The tribal populations in Jharkhand have rich cultural traditions, close ties to nature, and distinct social structures. However, they also face substantial socio-economic disadvantages, limited access to quality healthcare, and a growing burden of chronic diseases [4]. These realities make it essential to develop community-centric and culturally sensitive health interventions that address the emerging epidemic of hypertension among them.

Recent studies and data, including those from the National Family Health Survey (NFHS-5), have revealed startling trends. While the national pooled prevalence of hypertension is estimated at around 16.1%, certain tribal areas have reported much higher figures. For instance, in the hilly tribal regions of Kashmir, hypertension prevalence is as high as 41.4%. Gender-wise disaggregation shows that tribal men are more affected (46.7%) compared to women (37.9%) [5]. Such figures challenge the long-standing assumption that tribal communities are largely protected from lifestyle diseases and underscore the urgent need for targeted interventions.

One of the biggest challenges in managing hypertension in tribal areas is the issue of late diagnosis. Due to the silent nature of the disease, individuals rarely experience symptoms in the early stages. In tribal regions, where regular health check-ups are rare and healthcare infrastructure is sparse, many cases go undetected for years [4,5]. Often, hypertension is diagnosed only when individuals present with complications such as stroke, heart attack, or kidney failure. This delay in diagnosis leads to poorer outcomes and a heavier burden on the healthcare system. In states like Jharkhand, this is particularly problematic given the already high cause-specific mortality from stroke, which accounts for 14.3% of deaths [6]. Understanding the risk factors that contribute to this growing burden is essential. Lifestyle and behavioral factors are increasingly playing a role. Tribal communities, traditionally known for their active livelihoods and simple diets, are now experiencing rapid changes due to increased urban influence, market exposure, and sedentary occupations [7]. Surveys have shown that 46.1% use tobacco, and alcohol consumption is associated with tobacco use [8]. These substances are often consumed in unregulated forms, further compounding health risks. Diets have also undergone a transformation. With greater availability of packaged and processed foods, high in salt and low in essential nutrients, dietary risks have escalated, especially among young adults.

Another significant factor is the rise in obesity and physical inactivity. While once considered uncommon in tribal settings, sedentary lifestyles are now increasingly observed. A study by Kumar et al. reported that, in Jharkhand, overweight and obesity account for 38.4%, and abdominal obesity, a strong predictor of cardiovascular disease, is seen in 34.6% of the population [7]. These figures point to a major shift in the health profile of tribal communities. In addition, health literacy remains a critical barrier to effective hypertension management. With a literacy rate of 66.4% in Jharkhand (compared to the national average of 76.32%) [9,10], many individuals lack the basic knowledge needed to understand their condition, follow treatment protocols, or make informed health decisions. Even when patients receive prescriptions, follow-up is irregular, and medications are often discontinued midway. The burden of hypertension is not just clinical; it is also social and infrastructural. Jharkhand’s healthcare infrastructure is under significant strain. Although the state has a wide network of primary health centers (PHCs) and community health centers (CHCs), many are poorly equipped and understaffed [11]. Road connectivity is a serious challenge, especially in remote tribal areas. The state’s total road network includes just 1.39% of major roads. National highways account for only 2.3%, and state highways a mere 0.76% [3]. This makes transportation of patients, health workers, and essential supplies both time-consuming and expensive. Often, patients have to walk for hours or travel by limited public transport options to reach a health facility.

Cultural factors further complicate the scenario. Tribal communities have rich spiritual traditions and belief systems. Festivals such as Sarhul, Karma, and Sohrai are central to community life [12], and illness is often interpreted through spiritual or supernatural lenses. Many individuals initially seek help from local healers or spiritual guides before turning to modern medicine. While traditional systems have their strengths, such delays can be harmful in managing conditions like hypertension that require early and sustained intervention [13]. Any public health strategy, therefore, must recognize and respect these cultural beliefs while promoting evidence-based healthcare practices.

Economic limitations add another layer of difficulty. Jharkhand’s per capita government health expenditure stands at ₹801, which is less than half of the national average of ₹1,753 [3]. Out-of-pocket expenses deter many families from seeking or continuing care, especially for chronic conditions like hypertension, which require lifelong management. Despite these challenges, there is hope and progress. One of the most effective approaches is the community-based outreach model, where frontline health workers play a key role. Under the Ayushman Arogya Mandir model, screening, prevention, control, and management of non-communicable diseases will be provided at the community level [14]. Accredited Social Health Activists (ASHAs) are trained not only to take blood pressure but also to counsel patients, explain medication regimens, and conduct monthly follow-ups [15]. Their familiarity with the local language and culture makes them trusted figures in the community.

Another important development is the e-Sanjeevani telemedicine platform [16], which allows patients to consult doctors remotely. This is particularly useful in tribal areas where medical specialists are often not available locally. Through tele-consultation and e-prescriptions, patients receive advice, diagnosis, and treatment plans without having to travel long distances. This reduces costs, saves time, and ensures that more people are brought into the treatment net. A structured referral and counter-referral system has also been implemented. Once a patient is diagnosed with hypertension at the community or primary health center (PHC) level, they are referred to higher facilities such as a community health center (CHC) or district hospitals if needed. After stabilization and initial treatment, they are sent back to the PHC or community-level services for regular monitoring [17]. This ensures that patients receive the right level of care at the right time while also preventing unnecessary overcrowding at tertiary centers.

**Figure 1:**
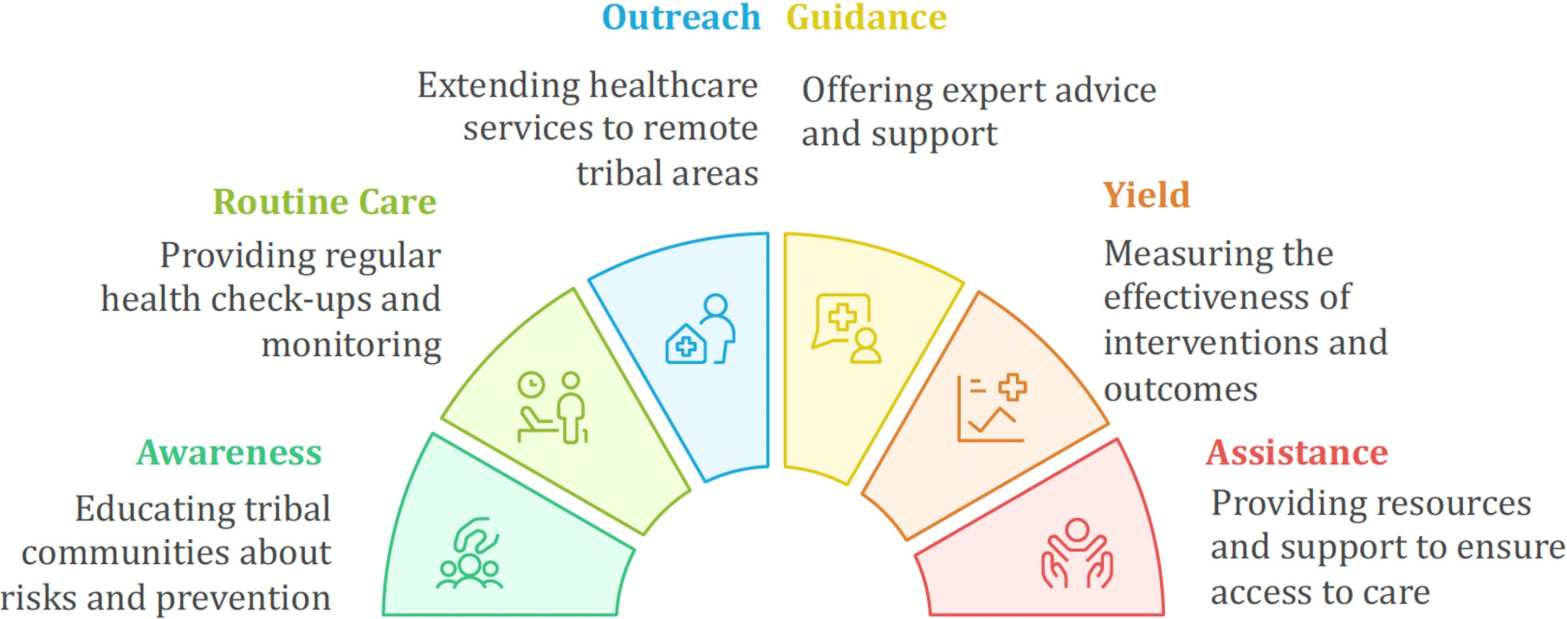
Hypertension Tribal Network for Accessible Awareness, Routine care, Outreach, Guidance, Yield, and Assistance framework Co-created by Dr. Himel Mondal and Napkin AI (https://www.napkin.ai) for this article

In our opinion, the following innovative strategies can be effectively implemented to enhance service delivery and reach underserved populations. For instance, during tribal festivals or special community events, mobile health units can be strategically deployed. Additionally, temporary outpatient clinics may be set up in accessible locations such as community halls or even individual homes, ensuring timely and culturally sensitive healthcare access. These events may offer health screenings, check-ups, and medication distribution, often with small incentives to encourage participation. Such models will not only improve access but also help integrate health services into the cultural rhythm of tribal life. To bring coherence to these diverse efforts, the concept of HTN-AAROGYA has been proposed. This stands for Hypertension Tribal Network for Accessible Awareness, Routine care, Outreach, Guidance, Yield, and Assistance (Figure 1).

The initiative seeks to build a structured yet flexible network of hypertension care that connects ASHAs, PHCs, telemedicine services, referral hospitals, and community leaders. The emphasis is on creating a sustainable ecosystem of care that is locally rooted and culturally sensitive.

One major challenge in managing chronic conditions like hypertension is preventing loss to follow-up. Patients may discontinue treatment for various reasons: migration for seasonal work, financial difficulties, or simply a lack of perceived need once symptoms subside. To address this, continuous engagement through follow-up visits, tele-consultations, and phone-based reminders is critical. Going forward, policy support and high-quality research will be key to sustaining and scaling these efforts. There is an urgent need to conduct a detailed STEPwise approach to NCD risk factor surveillance (STEPS) surveys focused on tribal populations to gather data on risk factors [18], healthcare utilization, and treatment outcomes. Such data will be invaluable for planning interventions, allocating resources, and tracking progress. It is also essential that future research be community-driven and sustainable [19]. Too often, tribal areas are subject to “helicopter research,” where external researchers conduct short-term studies without meaningful engagement or long-term impact [20]. Instead, research should involve local stakeholders, build local capacity, and address the real concerns of the community. Investments in infrastructure and capacity building must continue. Improving road connectivity, digital networks, and health facility standards can dramatically enhance service delivery. Training tribal youth as health workers or community educators not only creates employment but also builds trust in the health system. Health education materials should be developed in local languages and dialects, and traditional healers can be brought on board as allies in health promotion efforts [21].

In conclusion, managing hypertension in tribal areas like Jharkhand requires a multifaceted approach that blends medical science with cultural understanding, community empowerment, and technological innovation. Strengthening PHCs, leveraging digital tools like telemedicine, empowering frontline health workers, and integrating local customs into health outreach are all essential components. The HTN-AAROGYA framework offers a promising path forward by bringing together diverse elements into a coherent and adaptable system of care. By aligning public health initiatives with the unique realities of tribal communities, India has the opportunity to set an example in equitable, culturally sensitive, and sustainable healthcare. Only with such integrated and respectful efforts can we ensure that the battle against hypertension, indeed, against all NCDs, is won not just in cities and towns but in every remote village and tribal hamlet across the nation.
